# *Candida albicans* Agglutinin-Like Sequence (Als) Family Vignettes: A Review of Als Protein Structure and Function

**DOI:** 10.3389/fmicb.2016.00280

**Published:** 2016-03-15

**Authors:** Lois L. Hoyer, Ernesto Cota

**Affiliations:** ^1^Department of Pathobiology, University of Illinois at Urbana–Champaign, UrbanaIL, USA; ^2^Department of Life Sciences, Imperial College LondonLondon, UK

**Keywords:** fungus, *Candida albicans*, gene family, Als proteins, adhesion, aggregation, attachment, invasion

## Abstract

Approximately two decades have passed since the description of the first gene in the *Candida albicans* ALS (agglutinin-like sequence) family. Since that time, much has been learned about the composition of the family and the function of its encoded cell-surface glycoproteins. Solution of the structure of the Als adhesive domain provides the opportunity to evaluate the molecular basis for protein function. This review article is formatted as a series of fundamental questions and explores the diversity of the Als proteins, as well as their role in ligand binding, aggregative effects, and attachment to abiotic surfaces. Interaction of Als proteins with each other, their functional equivalence, and the effects of protein abundance on phenotypic conclusions are also examined. Structural features of Als proteins that may facilitate invasive function are considered. Conclusions that are firmly supported by the literature are presented while highlighting areas that require additional investigation to reveal basic features of the Als proteins, their relatedness to each other, and their roles in *C. albicans* biology.

## Setting the Scene

*Candida albicans* can exist in its human host as a commensal, and under certain circumstances, cause disease. *C. albicans* is the principal cause of opportunistic mycoses worldwide (Pfaller and Diekema, 2007). Adhesion is important for establishing the *C. albicans*-host interaction. The adhesive role of Als proteins stimulated enthusiasm for their study.

The first *ALS* gene, *ALS1*, was detected in a differential hybridization screen in the pre-genome era of *C. albicans* research (Hoyer et al., 1995). The protein was named because of its similarities to *Saccharomyces cerevisiae* alpha-agglutinin, which promotes cell–cell contact during mating (sexual reproduction) of haploid yeasts (Lipke et al., 1989). The presence in *C. albicans* of additional genomic fragments that hybridized with *ALS1* sequences suggested the existence of a gene family (Hoyer et al., 1995, 1998a). Additional effort revealed the full nature of the ALS family in *C. albicans* (Gaur and Klotz, 1997; Hoyer et al., 1998b; Hoyer and Hecht, 2000, 2001; Zhao et al., 2007a), which proved to be essential for accurate assembly of the *C. albicans* genome sequence (Braun et al., 2005). Cross-hybridization between *C. albicans ALS* sequences and genomic DNA from other *Candida* species suggested that similar genes are found in closely related fungi (Hoyer et al., 2001).

**FIGURE 1:**
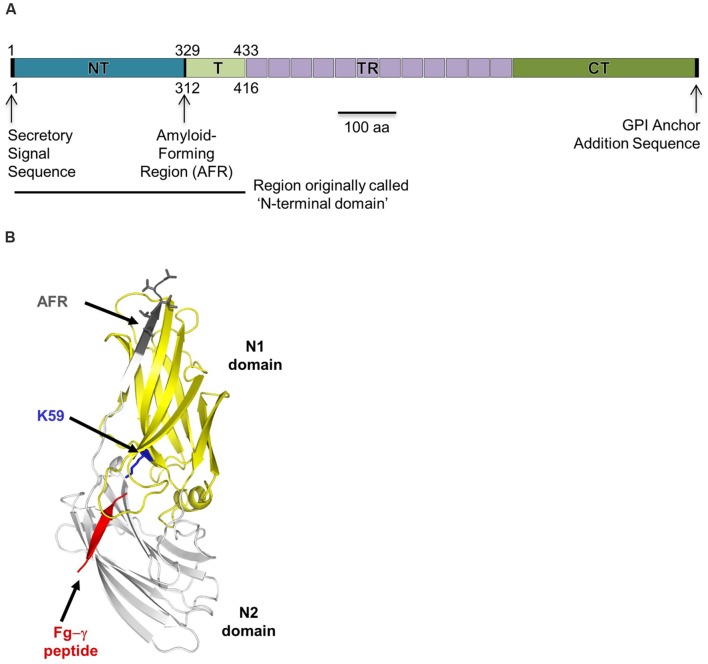
**Als protein structure. (A)** Line drawing of a representative Als protein, using *C. albicans* Als3 as the example. A detailed schematic comparing the basic features of all *C. albicans* Als proteins was published previously (Hoyer et al., 2008). The various domains are labeled as they are discussed in this review: NT (also called NT-Als), T, TR, and CT. Early literature referred to the sequences N-terminal of the TRs as the ‘NT’; this region is indicated by the solid line below the main drawing. Als proteins include a secretory signal sequence which is processed, so absent from the mature protein. Als proteins also encode a consensus sequence for GPI (glycosyl-phosphatidylinositol) anchor addition. The GPI anchor subsequently is processed and the mature protein linked to beta-1,6-glucan in the *C. albicans* cell wall (Kapteyn et al., 2000). Numbering schemes found in the literature may be confusing because some start at the initial Met (shown above the line drawing) while others start at the N-terminal amino acid of the mature protein, following cleavage of the secretory signal peptide (e.g., amino acid 18 of the unprocessed sequence in many of the Als proteins; shown below the line drawing). Clarifications are provided throughout the review to indicate whether the numbering scheme arises from the unprocessed (signal sequence present) or processed (cleaved signal sequence) protein. **(B)** X-ray crystallographic structure of the NT domain from Als9-2 in complex with the C-terminal peptide from fibrinogen-γ (red; Salgado et al., 2011) that fits into the protein’s PBC. An invariant Lys residue (K59, using a numbering scheme for the processed protein; blue) at the end of the PBC recognizes the C-terminal carboxyl group of the peptide ligand. The overall fold of the protein involves eight conserved Cys residues that form four disulfide bonds. In the ligand-bound form of the protein, the AFR (gray) attaches to the NT-Als surface. The AFR is unattached to the NT-Als surface in protein molecules that do not have a ligand in the PBC.

The novelty of coding tandem repeats in *ALS* genes figured largely into initial conceptual thinking about organization of the genes and their encoded proteins. For example, early descriptions of a typical Als protein reported three domains: the central tandem repeats, everything before the repeats (N-terminal domain), and everything after the repeats (C-terminal domain; **Figure 1A**). As investigations proceeded, Als proteins were described as including four domains: the N-terminal domain (NT or NT-Als; approximately amino acids 1–329 of the unprocessed protein), the T domain (T; approximately amino acids 330–433, ending just at the start of the tandem repeats), the central tandem repeats (TR), and the CT. Two notable sequence features prompted the idea that the NT and T domains should be considered separately: the Thr richness of amino acids 330–433 and the presence of a short sequence that has amyloid-forming propensity (approximately amino acids 325–329; Garcia et al., 2011). However, crystallographic analysis demonstrated that the AFR is a part of the NT structure (Salgado et al., 2011; Lin et al., 2014; **Figure 1B**), leaving open for question the best way to describe the domains of a typical Als protein. Because many manuscripts use the four-domain description of Als proteins, that convention is featured in **Figure 1** and throughout this review.

Over the years, as cell-biological observations about the Als family accumulated, the *C. albicans* research field also matured, providing new reagents and approaches for studying the Als family, as well as growing knowledge about numerous aspects of *C. albicans*–host interactions. Availability of the crystallographic structure of NT-Als (Salgado et al., 2011; Lin et al., 2014; **Figure 1B**) allows examination of the structural basis for Als function. This review manuscript critically interprets the literature in light of recent structural insights, as well as the abundance of new fungal genome sequences. The review is configured as a set of questions (vignettes) that focus on various properties of the Als proteins. Therefore, the review does not contain an exhaustive list of Als-related literature, but instead focuses on selected reports that shape the answer to the overall question “What do we really know about Als proteins and the mechanistic basis for their function?”

## What Genes/Proteins are Included in the *Als*/Als Family?

When *C. albicans ALS1* was first described, a BLAST search of the non-redundant protein database produced one ‘hit’: *S. cerevisiae* alpha-agglutinin (now named Sag1; Lipke et al., 1989). We now know that there are many *ALS* genes in *C. albicans* and that *ALS* genes are present in other fungal species. Moreover, recent structural biology insights, coupled with long-standing functional observations, raise the question of whether Sag1 belongs to the Als family. These topics are reviewed here with the goal of defining the minimum features that are needed for including a protein in the Als family. Overall, the observations suggest that the Als family is more diverse than currently envisioned.

The composition of the ALS family is most clear for *C. albicans*, in which eight distinct loci are known (*ALS1* to *ALS7*, and *ALS9*; Hoyer et al., 2008). *ALS* gene names were assigned sequentially as the genes were discovered. Recombination between two contiguous loci (*ALS5* and *ALS1*) led to production of a novel open reading frame (ORF) in some strains; this locus was named *ALS51* to indicate its chimeric origin (Zhao et al., 2011). Each *ALS* locus encodes numerous alleles, with sequence variation occurring primarily in the tandem repeat and C-terminal domains (Zhang et al., 2003; Zhao et al., 2003, 2007c; Oh et al., 2005). Many sequence variants encoding the NT domain of Als5 have also been documented (Zhao et al., 2007c); such NT sequence variation may exist for other Als proteins, as well. Allelic variation caused confusion in providing a name for *ALS8*, which proved to be the same physical locus as *ALS3* (Zhao et al., 2004).

Based on *C. albicans* sequences, the consensus definition of Als proteins includes those with an NT domain, followed by the T, TR, and CT sequences (NT/T/TR/CT). The secretory signal peptide and GPI anchor addition sequence are key features that direct mature proteins to their localization in the cell wall, so also should be included in the consensus definition. The NT domain of Als proteins encodes conserved Cys residues that are key for folding of the protein, as well as the invariant positively charged amino acid (e.g., Lys59 in NT-Als3; Lin et al., 2014) located at the end of the PBC. These generalizations hold true for Als proteins in *Candida dubliniensis*, perhaps the closest relative of *C. albicans*. Sequence similarities and synteny analysis revealed that *C. dubliniensis* includes all *C. albicans* Als proteins except Als3 and Als5 (Jackson et al., 2009). *C. dubliniensis* has an extra Als protein that is not syntenic with those in *C. albicans*.

Unlike the initial BLAST search many years ago, a current BLAST search yields dozens of ‘hits,’ fueled by the availability of numerous fungal genome sequences. The sequence data provide a catalog of potential Als proteins much more readily than previous laborious methods such as cross-hybridization studies and amplification of sequences using degenerate primers (Hoyer et al., 2001). Butler et al. (2009) presented the best-known analysis of the Als family from the perspective of multiple fungal genomes. The study includes pathogens (*Candida tropicalis, Candida parapsilosis, Meyerozyma* (*Candida*) *guilliermondii, Clavispora* (*Candida*) *lusitaniae*) and non-pathogenic species (*Lodderomyces elongisporus, Debaryomyces hansenii*). Characterization of Als protein function in the pathogenic species is emerging, showing a role in adhesion and pathogenesis similar to the Als proteins in *C. albicans* (Bertini et al., 2015). The genomes of fungi that are important in biofuel production have been sequenced (e.g., *Scheffersomyces (Pichia) stipitis, Candida tenuis, Spathaspora passalidarum*) and Als proteins are predicted in them (Jeffries et al., 2007; Wohlbach et al., 2011; Maguire et al., 2013). Because Als proteins in these species are unlikely to mediate interactions with a mammalian host, studies to examine their function will provide novel insights.

As genome sequencing efforts advance, sequences are available for an ever-larger number of fungi (Grigoriev et al., 2014), as well as multiple isolates from the same species, providing the opportunity to compare strain diversity (Pryszcz et al., 2015). However, the rapid accumulation of fungal sequence data has outpaced the ability to refine genomic assemblies. Because *ALS* genes often contain extensive tracts of tandemly repeated sequences, they are extremely difficult to assemble correctly using automated methods. This same problem existed for *C. albicans*: accurate assembly of the *ALS* genes relied heavily upon laboratory experimentation to define the ORFs and corresponding physical loci (Braun et al., 2005). Descriptions of current *ALS* sequences derived from genome data provide the impression that the analysis is very precise, however, closer examination reveals incompletely assembled and misassembled ORFs that are not a solid foundation for such detailed conclusions. ORFs identified as unique sometimes lack a 5′ end or a 3′ end. Some ORFs lack both, existing only as tracts of *ALS*-like tandem repeats that are not joined to anything else. In most genome sequences, considerable effort will be required to answer even the most basic questions such as how many *ALS* loci are present.

Despite the need for follow-up experimentation, the genome sequence data provide sufficient information to indicate that while fungal species encode NT/T/TR/CT ALS genes like those in *C. albicans*, other variations exist. For example, some species have *ALS* genes with novel TR sequences of varying unit length and composition. Some of the fungal genomes encode at least one NT/T/TR/CT Als protein and one that includes only NT and CT, suggesting the potential need to redefine the minimum features for including a gene in the ALS family. The NT/CT gene structure is more akin to *S. cerevisiae* Sag1 than *C. albicans* Als proteins (**Figure 1**). Closer examination of the NT domain of these proteins reveals a sequence that perhaps is also more like Sag1. The NT of Sag1 is predicted to include two immunoglobulin domains with three of the four disulfide bonds that are present in NT-Als (Grigorescu et al., 2000; Salgado et al., 2011). Functional analysis of Sag1 revealed that it binds the free C-terminal peptide of **a**-agglutinin, Aga2 (Cappellaro et al., 1994). Sag1 and other Sag1-like proteins include a positively charged amino acid (Arg) in a structurally equivalent position to the Lys residue located at the end of the PBC, suggesting that they also contain this cavity (Cota and Hoyer, 2015). Sag1 has resisted efforts to solve its structure (Grigorescu et al., 2000) so structural data are not available for comparison.

Sequence similarities and predicted structural similarities between Als and Sag1-like proteins raise the question of whether to consider them as part of the same family. Evidence to support the idea that the proteins belong to the same family includes the fact that the sequences share sufficient similarity to recognize each other using a simple BLAST search. However, while Als and Sag1-like proteins are also predicted to share similar structural features, they represent two functional ‘extremes.’ Als protein NT domains mediate adhesion to a broad range of ligands with moderate-to-low binding affinities, which facilitates their role in host-pathogen interaction (Donohue et al., 2011; Salgado et al., 2011). In contrast, the NT domain of Sag1-like proteins mediates cell–cell contact during the mating of haploid cells, and therefore ligand recognition must be very selective to maintain the integrity of the mating interaction. Binding between Sag1 and the C-terminal peptide of Aga2 occurs at a high affinity (Zhao et al., 2001). This separation between the proteins suggests that perhaps it is more appropriate to consider Als and Sag as different families, closely related by their similarities in sequence and structure. As new genome data emerge and functional analyses progress, it will become clear whether other Als and Sag proteins exist at the functional extremes described above or whether intermediate proteins exist (e.g., an Als-like protein with high affinity for a limited number of ligands). These data will provide additional evidence regarding classification of the overall group of proteins.

Another point to resolve in defining the ALS family across various fungal species is to assign a name for each gene. The Butler laboratory’s extensive analysis of synteny between various fungal genomes provides a starting point for this discussion. The synteny analysis initially focused on two strains of *C. albicans* and a single isolate each of eight other species (Fitzpatrick et al., 2010), then was expanded to include a total of 13 species (Maguire et al., 2013). Results of the analysis are visualized easily using the Candida Gene Order Browser (http://cgob.ucd.ie) that highlights syntenic loci that are perhaps the most deserving of a name that is the same as a *C. albicans ALS* gene. However, conservation of protein function cannot be assumed from syntenic genes (Jackson et al., 2009) and regulation of syntenic genes may vary between species. Northern blot analysis of total RNA isolated from several different growth conditions suggested that *C. dubliniensis ALS* ORFs tend to be constitutively expressed, in contrast with the differential expression noted for many *C. albicans ALS* genes (Hoyer et al., 2001). Fortunately, most publications refer to *ALS* genes by the ID numbers assigned by the genome sequencing effort (e.g., Bertini et al., 2015). This practice avoids confusion by providing unambiguous reference to specific loci. Indeed, even though the literature appears to include considerable information about *C. albicans* Als proteins, their true functional relatedness is still relatively unexplored and the phenotypes observed may be heavily influenced by protein abundance and localization on the fungal cell surface rather than the true functional capabilities of the protein. Much of this review article examines these questions.

The list of *ALS* genes and Als proteins is certain to become longer and more diverse with the emergence of new genome sequencing data and functional insights. The current definition of Als proteins including the NT/T/TR/CT configuration will likely need to be broadened. Perhaps the minimum definition of an Als protein will only include the NT domain and sufficient structure to display it on the fungal cell surface. This definition would place more emphasis on protein function than on absolute number and configuration of Als domains. The ligand-binding activity of Als proteins is perhaps their most important function and is examined in detail in the subsequent section.

## How Do Als Proteins Bind Ligands?

The adhesive function of Als proteins is a major reason for studying them. Adhesion is an important feature of colonization, which provides the potential for disease development (Calderone and Braun, 1991). While it might appear easy to define the word ‘adhesion,’ its liberal use in the literature describes many different interactions. This diversity of interactions makes a precise definition elusive, especially for a molecule like an Als protein that has many different sticky interactions. For example, the process of Als proteins binding to each other might be called ‘adhesion’ in the literature, but could be more precisely described as ‘aggregation.’ Likewise, ‘attachment’ may be a better word choice to describe the non-specific interactions between *C. albicans* and abiotic surfaces (discussed below). Here, we define ‘adhesion’ as ligand binding with the goal of exploring the molecular mechanisms and structural features of the Als protein that are involved in this process.

Als proteins were demonstrated to function in adhesion by deletion of *ALS* genes from *C. albicans* or expression of *ALS* genes in *S. cerevisiae*, leading to reduction or gain of adhesive function, respectively (reviewed in Hoyer et al., 2008). Because deletion of *ALS3* provides the greatest loss of adhesive function among the ALS family, it gained considerable attention in the literature. Cell biology-based inquiry provided an extensive list of divergent binding partners for Als3 including human fibronectin, laminin, collagen, gp96, EGFR, HER2, N-cadherin, E-cadherin, fibrinogen, casein, equine ferritin, bovine serum albumin (BSA), and *Streptococcus gordonii* SspB (Gaur et al., 2002; Gaur and Klotz, 2004; Sheppard et al., 2004; Phan et al., 2007; Almeida et al., 2008; Silverman et al., 2010; Liu et al., 2011; Zhu et al., 2012). The NT domain was implicated in much of the inferred protein–protein interactions (Loza et al., 2004; Sheppard et al., 2004; Zhao et al., 2006). Molecular modeling was used to conclude that the Als3 NT domain interacts with its binding partners by surface–surface interactions (Sheppard et al., 2004; Phan et al., 2007). However, the large number of proposed binding partners raises the question of how NT-Als can adapt to surfaces of so many structurally unrelated ligands to mediate relevant interactions. Solution of the molecular structures of the NT domains of Als1, Als3, and Als9-2 (Salgado et al., 2011; Lin et al., 2014) provided an Als protein model with atomic resolution that can reconcile these observations (**Figure 1B**). A discussion of how NT-Als structural data inform functional insights was communicated recently (Cota and Hoyer, 2015) and is presented briefly here.

The overall NT-Als fold is reminiscent of bacterial adhesins such as *Staphylococcus aureus* ClfA (clumping factor; Deivanayagam et al., 2002) and *Staphylococcus epidermidis* SdrG (Ponnuraj et al., 2003). Unlike the bacterial proteins, however, NT-Als contains a wide and flat cavity (PBC) between domains that can bury up to six C-terminal residues of peptides in an extended conformation (**Figure 1B**). The side chain amine of an invariant Lys at the end of this cavity (K59) establishes a salt-bridge with the C-terminal carboxylic acid of the incoming peptide. The peptide backbone forms extensive hydrogen bonds in parallel orientation to a β-strand (G2) from the second Ig domain. Water molecules mediate interaction with the A2 strand on the other side of the peptide. Water molecule number and arrangement are variable depending on the peptide ligand, and provide the ability for NT-Als to recognize a broad array of ligands. Thus, NT-Als has a novel mechanism to bind the flexible C terminus of proteins.

**FIGURE 2:**
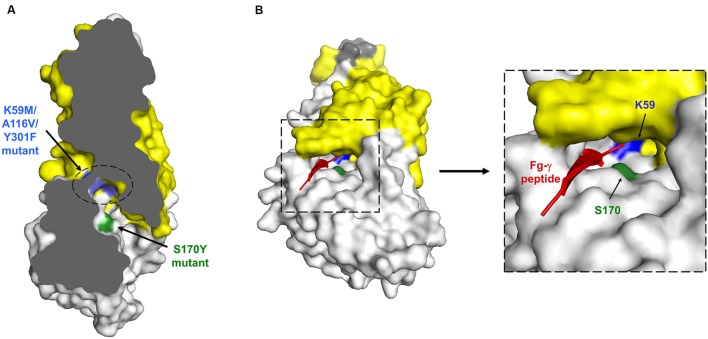
**Schematics of NT-Als protein structure to illustrate the location of mutations used to deduce the ligand-binding mechanism. (A)** Cross-section of overall NT-Als3 structure highlighting the location of the PBC and key residues used in loss-of-function mutants. Note that the indicated mutations were introduced without altering NT-Als surface properties. Amino acid numbering reflects the processed (signal peptide removed) form of the protein (Lin et al., 2014). **(B)** Expanded PBC detail showing entry of a model peptide and location of amino acids included in the functional analysis using the structure of NT-Als9-2 (Salgado et al., 2011).

Als3 was selected as a model for mutational analysis to test structural hypotheses in a native *C. albicans* background (Lin et al., 2014). Mutations were designed to interfere with PBC function and also with function of the AFR that is located within the NT-Als domain. The role of the AFR in Als-mediated aggregation is discussed extensively in the literature and examined in subsequent sections below. PBC mutations involved altering either three amino acids (K59M, A116V, Y301F) or one amino acid (S170Y; **Figure 2**). The resulting mutations did not change the surface structure of Als3 and mutant proteins lacked peptide-binding capabilities *in vitro.* Mutations were made in *ALS3* constructs that were integrated into the *ALS3* locus of a *C. albicans Δals3/Δals3* strain. Immunolabeling with an Als3-specific monoclonal antibody (Coleman et al., 2009) demonstrated the presence of Als3 on the *C. albicans* surface in comparable quantities and location to those produced by a wild-type-Als3 control construct. The adhesive phenotype of the resulting strains was absolutely remarkable: strains with the targeted mutations had the adhesive phenotype of a null mutant strain in standard adhesion assays involving complex surfaces such as cultured and fresh human cells (Lin et al., 2014), as well as whole bacterial cells (*Streptococcus gordonii*; Hoyer et al., 2014). In other words, alteration of one or three amino acids and display of the mutant protein on *C. albicans* resulted in the same phenotype as a strain on which no Als3 was displayed at all.

Collectively, these data provided a striking demonstration of the importance of the PBC to Als ligand-binding activity and its overall contribution to the phenotypes observed in standard adhesion assays. The idea that the PBC binds the free C-terminal end of proteins was reinforced because of the use in structural analyses of small synthetic peptides that readily fit into the PBC in that orientation. However, the mechanistic conclusion is also satisfying because of the strong conservation of K59 among *C. albicans* Als sequences and similar proteins identified in database searches. The positive charge of K59, positioned at the bottom of the PBC, is available to sink the net negative charge of the carboxyl end of a C-terminal peptide. Although purified proteins were key to deducing this mechanism, they provide a much more simple set of interactions than those involving whole Als proteins and a complex cell surface. Testing of the *C. albicans* PBC mutant strains with whole *Streptococcus gordonii* cells supported previously published work that showed SspB is an Als3 binding partner (Silverman et al., 2010; Hoyer et al., 2014). However, the C terminus of SspB is covalently linked to the bacterial cell wall, suggesting that Als3 must recognize other sequences in the large cell-surface SspB. Proteolytic action to create adhesion tethers (SspB-derived free C-terminal peptides that remain attached to SspB) was proposed as one hypothesis to explain the observations (Hoyer et al., 2014; Cota and Hoyer, 2015). Other hypotheses also exist and suggest that while considerable progress has been made toward understanding the mechanism by which Als proteins bind ligands, additional puzzles remain to be solved.

A discussion of the Als ligand-binding mechanism would not be complete without addressing the widespread notion that Als proteins also function as lectins, recognizing carbohydrate ligands. This information comes from multiple sources. Some sources may contain simple errors. Some may have improperly drawn conclusions from BLAST search data that reflected amino acid compositional similarities, rather than conservation of function. One experimental report exists that concludes a role for Als1 in binding fucose. These observations are discussed here.

BLAST searches are widely used because they provide quick information about the potential function of a newly characterized protein. A BLAST search of the non-redundant protein database using a *C. albicans* NT-Als sequence as the query leads to an alert for detection of a putative conserved domain called ‘Candida_ALS_N superfamily.’ Clicking on the alert leads the reader to this statement: ‘This is likely to be the sugar or ligand binding domain of the yeast alpha-agglutinins.’ This statement is problematic because it suggests lectin activity as the primary role for each protein, which is not true for either Als proteins or Sag1.

Databases within the *Candida* community, such as the Candida Genome Database (CGD; Inglis et al., 2012) also contribute to the conclusion that Als proteins bind carbohydrates. *C. albicans ALS* loci in CGD are appropriately annotated to reflect their protein-binding function, however, data for other *Candida* species are not developed fully and contain misleading information. *C. dubliniensis* locus Cd36_64220 is a useful example because it is syntenic with *C. albicans ALS9*. As expected, *C. albicans ALS9* is the best match for Cd36_64220 in a BLAST search. It would be reasonable to hypothesize that the proteins have similar function. However, *S. cerevisiae FLO1*, a large cell-wall lectin that binds mannose (Veelders et al., 2010) is listed as an ortholog for Cd36_64220. The Ser/Thr-richness and extensive tracts of repeated sequences in both Als proteins and Flo1 are likely responsible for these database searching ‘hits’ that are distractions, rather than indications of similar function.

One literature report suggests a role for Als1 in recognition of carbohydrate ligands (Donohue et al., 2011). The authors constructed a *S. cerevisiae* strain that secreted a soluble hexa-His-tagged NT/T Als1 protein. They applied the Als protein fragment to a glycan array and detected it with an anti-His antibody. Fucose-containing glycans were preferentially recognized. Fucosylated BSA was used in subsequent experiments to calculate the affinity of the interaction, but a BSA-alone control was not tested. Because Als proteins are known to recognize BSA (Klotz et al., 2004), the interaction between fucosylated BSA and the Als1 fragment may have indicated the affinity of the Als1–BSA interaction rather than the Als1–fucose interaction. Although the glycan array results suggest the possibility that the NT/T portion of Als1 can bind fucose, the mechanistic basis for this interaction remains unexplored. PBC involvement could be tested using structurally informed mutant proteins (Lin et al., 2014) and appropriate controls to support the conclusion of carbohydrate binding. The availability of structural data provides the opportunity to describe Als ligand-binding function at the molecular level. The abundance of published data that describe a role for Als proteins in peptide binding suggest that this function will outweigh any potential lectin activity and should be listed as the primary Als function in various reference databases.

## Do Als Proteins Mediate Attachment to Abiotic Surfaces?

Questions about attachment of *C. albicans* to abiotic materials arise from a practical standpoint: *C. albicans* is able to form biofilms on the surface of implanted medical devices and attachment is an important initial step in biofilm formation. In addition to the role of Als proteins in binding peptide ligands, literature reports suggest that Als proteins are important for *C. albicans* attachment to abiotic surfaces. Although, this conclusion appears widely accepted, the mechanisms involved are still unclear. It is informative to separate the contribution of Als proteins to this function because many other cell wall proteins on the *C. albicans* surface [i.e., containing hydrophobic/amyloidogenic or glycosylated regions (Ramsook et al., 2010; de Groot et al., 2013)] could promote the same behavior. Several manuscripts were selected to represent the major viewpoints in this discussion (**Table 1**) and are detailed below.

**Table 1:** Summary of key features from three published manuscripts that describe a role for Als proteins in attachment of *Candida albicans* to abiotic surfaces.

Manuscript	Aoki et al., 2012	Finkel et al., 2012	Garcia et al., 2011
Yeast strain	*Saccharomyces cerevisiae* producing cell-surface Als NT/T/FLAG tag/alpha-agglutinin fusion proteins; one made for each Als protein	Wild-type Als proteins present on *C. albicans* cells grown to saturation in YPD, then released into fresh YPD; high levels of cell-surface Als1	*S. cerevisiae* overproducing Als5
Abiotic surface	Borosilicate glass, polypropylene, polyvinylchloride, polyurethane, polymethyl methacrylate, polytetrafluoroethylene, titanium	Fluxion flow chamber	Non-tissue-culture-treated polystyrene
Assay conditions	Cells washed and suspended in PBS	Cells resuspended in YPD	Cells washed and resuspended in TE
Quantification of adhesion	Plate 6M urea wash and count colonies	Capture image and count adherent cells	Absorbance at 570 nm to quantify retained crystal violet dye
Conclusion(s)	Yeast cells adhered to polypropylene, polyvinyl chloride and borosilicate glass, but not the other materials	*C. albicans* binds to PDMS channels but not to borosilicate glass; Als1 implicated by testing null mutant strain	Als5 promotes adhesion to polystyrene
Proposed adhesive mechanism	Ruled out hydrophobicity; implicated ‘substrate recognition pockets’	Not specified	Amyloid-forming region
Other mechanisms to consider?	Non-specific protein adsorption	Non-specific adsorption of YPD proteins to silicone surface, followed by PBC-mediated Als adhesion	Hydrophobic interactions

*YPD, yeast extract-peptone-dextrose medium; PBS, phosphate-buffered saline; TE, Tris-EDTA; PDMS, polydimethylsiloxane.*

### The Observations

Work by Aoki et al. (2012) has been cited as evidence that Als proteins mediate attachment to abiotic surfaces (de Groot et al., 2013; Demuyser et al., 2014). The authors used a constitutive promoter to drive production of a *S. cerevisiae* cell-surface fusion protein consisting of the NT/T region of Als proteins on a stalk composed of the C-terminal half of alpha-agglutinin. Fusion-protein-displaying yeast cells attached to polypropylene and polyvinyl chloride plastics, as well as borosilicate glass. No attachment was observed to polyurethane, polymethyl methacrylate, polytetrafluoroethylene, or titanium.

Finkel et al. (2012) sought to understand transcriptional regulation of *C. albicans* attachment to channels in a Fluxion flow cell, which has a borosilicate glass floor and polydimethylsiloxane (PDMS; silicone) walls. The authors observed that *C. albicans* attaches to the PDMS walls in the flow cells, but not to the borosilicate glass floor. A *bcr1/bcr1* strain showed reduced attachment to PDMS under flow conditions; testing of Bcr1 targets revealed attachment defects for an *als1/als1* strain. *C. albicans* growth conditions used for these assays produce high levels of Als1, but not Als3 (Coleman et al., 2010). This work suggested a role for Als1 in *C. albicans* attachment to PDMS.

Garcia et al. (2011) overproduced Als5 on the *S. cerevisiae* surface and evaluated the strain for biofilm formation in a polystyrene dish; conclusions were also drawn regarding the role of Als5 in attachment to polystyrene. Attachment was quantified by measuring crystal violet retained in each assay well. Micrographs were also captured. Micrographs showed greater numbers of attached cells for the Als5-producing strain compared to an empty vector control strain. These results suggested a role for Als5 in attachment to polystyrene.

### Attachment Mechanisms Proposed by the Authors

Aoki et al. (2012) used an assay that partitioned cells between water and *n*-octane to estimate hydrophobicity of the recombinant *S. cerevisiae* strains expressing Als fragments. Because they did not detect a positive correlation between these measurements and attachment data, the authors ruled out hydrophobicity as a potential mechanism for Als-mediated attachment. The authors concluded ‘that ALS proteins bound to the abiotic surfaces mainly by a specific adhesion mechanism between the material and the substrate recognition pockets of the ALS proteins’ although it is unclear how this interaction would occur. Finkel et al. (2012) implied that Als1 was directly involved in attachment to PDMS, but did not propose an attachment mechanism.

Garcia et al. (2011) suggested that the Als5 AFR (-IVIVATT-) is ‘critical for… cell-substrate adhesion to polystyrene.’ Their conclusion was based on decreased crystal violet retention by *S. cerevisiae* cells producing wild-type Als5 compared to cells producing an Als5 variant in which the AFR was mutated (-INIVATT-). However, micrographs show clearly that AFR mutation decreases cellular aggregation and overall cell abundance in the assay well. In other words, fewer cells are present in the well because of the reduction in the number of aggregated cells, rather than a reduction in the number of cells directly attached to polystyrene. Counting cells that are in direct contact with the polystyrene, rather than quantifying attachment using crystal violet (which cannot distinguish attachment from aggregation), could resolve these relationships.

### Could Hydrophobicity Be Involved in Als Attachment to Abiotic Surfaces?

Hydrophobicity has been invoked as a general property of Als proteins and bears additional discussion because of its potential to influence Als-mediated attachment to abiotic surfaces. Certainly, anyone who has ever attempted to collect *C. albicans* germ tubes by centrifugation has witnessed multiple phenomena (e.g., cellular aggregation, adsorption to the plastic tube, resistance to sedimentation) that could be attributable in part to hydrophobicity. Different regions conserved in the Als architecture could promote these interactions.

Frank et al. (2010) used molecular modeling approaches to conclude that the Als tandem repeat units (TRs) have both hydrophilic (contributed by glycosylation) and hydrophobic (due to predicted exposed patches of amino acids) components. The authors hypothesized that the hydrophobic nature of the TRs allows Als5 to mediate binding to polystyrene. Beaussart et al. (2012) showed the hydrophobic character of the *C. albicans* germ tube surface by probing it with an AFM tip that was functionalized with hydrophobic groups. Compared to wild-type *C. albicans*, an *als1/als1 als3/als3* null strain had a significantly decreased interaction with the hydrophobic AFM tip. Cells analyzed in this study were required to stick to a hydrophobic surface, however, perhaps introducing biases in measurement. Overall, though, these observations suggest contributions of the Als proteins to hydrophobicity in the context of otherwise ‘sticky’ germ tubes. Because polystyrene is very hydrophobic (e.g., Curtis et al., 1983; Ryan, 2008), hydrophobicity may contribute to the observation that Als5 is involved in attachment to this surface (Garcia et al., 2011).

Investigations into the relationship between CSH and *C. albicans* attachment to polystyrene are not new for the field and served as a major focus for the laboratory of Kevin Hazen in the 1980s. CSH was initially investigated using a water/hydrocarbon partitioning assay until a polystyrene microsphere adhesion assay was developed to evaluate CSH of individual cells (Hazen and Hazen, 1987). Revisiting this literature in the context of current knowledge of cell surface localization and abundance of Als proteins yields striking parallels: the overall relative changes in CSH observed with different growth phases and growth media are highly similar to the abundance and localization of Als proteins on the *C. albicans* cell surface. For example, transfer of yeast cells from a saturated culture into fresh growth medium resulted in a sharp rise in CSH (Hazen and Hazen, 1988). These growth conditions produce large quantities of Als1 on the yeast cell surface (Coleman et al., 2010). Similarly, CSH is higher for *C. albicans* yeast cells grown at room temperature compared to 37°C; lower growth temperatures promote greater cell-surface quantities of Als4 (Coleman et al., 2012). Germ tubes are more hydrophobic than yeast cells, and they tend to be far more homogeneously coated in Als proteins than individual yeast cells in a culture population (Hazen et al., 1986; Coleman et al., 2009, 2010, 2012). While positive correlation does not necessarily indicate cause-and-effect, the relationship between Als protein localization and abundance and CSH of those cell types is consistent with the idea that Als proteins contribute to *C. albicans* CSH and, therefore, to attachment to abiotic surfaces.

### Other Mechanisms to Consider

Other interactions besides hydrophobicity could also contribute to the interactions between Als proteins and abiotic surfaces. Although the idea has not appeared in any published manuscripts, non-specific protein adsorption to solid surfaces may be involved in these interactions. An extensive literature exists discussing non-specific factors that mediate protein adsorption onto solid surfaces (e.g., Hlady and Buijs, 1996; Goebel-Stengel et al., 2011). Non-specific protein adsorption to solid surfaces could contribute to the observations of Aoki et al. (2012).

Specific interactions between the Als PBC and adsorbed proteins may also explain some of the published observations. For example, the work of Finkel et al. (2012) was conducted in YPD medium, which contains an abundance of protein fragments. Such proteins could efficiently coat surfaces like PDMS and provide anchoring points for the Als1 PBC, accounting for at least a part of the observed phenotype. This work would parallel introduction of medical devices into the body. Upon exposure to fluids such as serum or saliva, abiotic surfaces would quickly become coated with soluble proteins, such as serum albumin (Hawser and Islam, 1998) or salivary statherin (Johansson et al., 2000). PBC activity may work in conjunction with other mechanisms, but unlike hydrophobic/glycosylated contacts, it has the potential to provide specificity to the initial association of *C. albicans* with surfaces of different chemical compositions. Nonetheless, interaction with soluble proteins could also impair attachment to these materials, modulating the association of *C. albicans* with the host surfaces, the microbiota and different factors of the immune system. The interplay and relevance of these binding mechanisms remain to be characterized.

### Are All Als Proteins Equal in Attachment to Abiotic Surfaces?

Although the mechanism(s) of Als protein attachment to abiotic surfaces require(s) additional investigation, we can speculate whether these properties are unique to a subset of Als proteins or shared across the family. Aoki et al. (2012) tested each of the Als proteins and concluded that ‘most of’ them bound polypropylene, polyvinyl chloride and borosilicate glass. Finkel et al. (2012) tested null mutant *C. albicans* strains and implicated Als1 in adhesion to silicone, but ruled out Als3 because the null mutant failed to affect attachment in the flow assay. It is not surprising that Als3 failed to be implicated in attachment to silicone, because *C. albicans* does not produce Als3 under the growth conditions studied. Given the considerable similarity between Als1 and Als3 at the primary sequence level (Lin et al., 2014), it is likely that Als1 and Als3 have comparable silicone-attachment properties. Garcia et al. (2011) observed that wild-type *C. albicans* attaches to polystyrene, and commented on the potential for other Als proteins to show similar characteristics to those observed for Als5. Their explanation focused on identity between amyloid-forming sequences in the various Als proteins, leading to the conclusion that Als1 is likely to attach to polystyrene. However, the growth conditions used for the assay favor Als4 abundance on the *C. albicans* surface (Coleman et al., 2012). If attachment to abiotic surfaces involves a property that is common to the Als proteins (e.g., TRs), it is likely that they all may exhibit similar function. The availability of Als structural data and attribution of function to various Als structural features permits a mechanistic dissection and an understanding of what appear to be multiple factors that contribute to Als-mediated interaction with abiotic surfaces.

## Do Als Proteins Interact with Each Other?

There are many literature reports that describe Als–Als interactions as the basis for *C. albicans* phenotypes important for colonization and subsequent pathogenesis. Here, we review some of these examples, with an emphasis on examining the mechanistic basis for the interaction between Als molecules.

**FIGURE 3:**
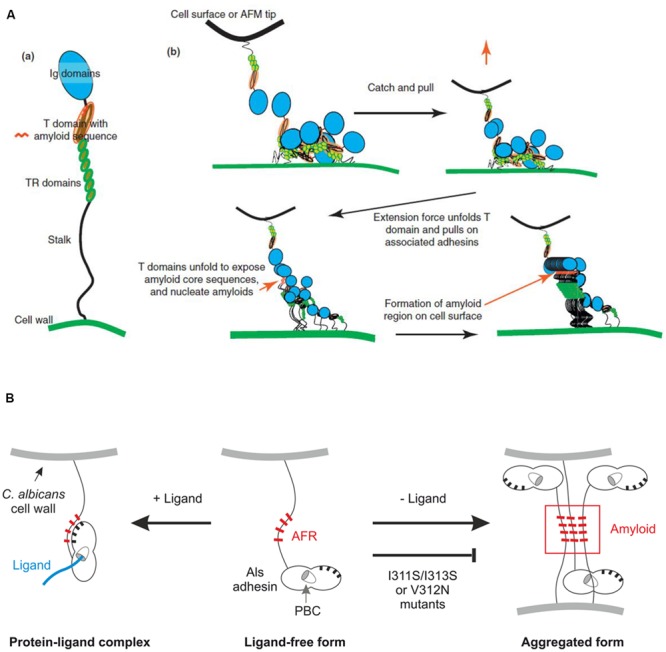
**Models proposed to explain function of the AFR in Als protein interactions. (A)** Force-induced aggregation of Als proteins on the surface of the same cell from Lipke et al. (2012). Homotypic binding between NT domains of Als proteins is proposed to trigger force required to pull apart an Als protein, exposing the AFR for interaction with AFR sequences on other Als proteins. **(B)** Variable conformation of the AFR in relation to the NT domain of Als3 on the *C. albicans* cell surface based on Lin et al. (2014). Newly synthesized Als protein can either bind ligand via the PBC, which results in the AFR attaching to the NT domain surface (left) or use its free AFR to interact with others, forming protein and cellular aggregates (right). Note that the model in **(A)** and the model in **(B)** show different artistic interpretations of AFR placement, with **(B)** showing an exaggerated scale of the NT portion of the molecule (especially the AFR) to emphasize those interactions. **(A)** Reprinted from Lipke et al. (2012), with permission from Elsevier. **(B)** This research was originally published in Lin et al. (2014). Reprinted with permission from The American Society for Biochemistry and Molecular Biology.

Several manuscripts suggest that Als homotypic binding is mediated by the NT domain. Perhaps these first arose through hypotheses regarding the interaction of Als proteins with cadherins (Phan et al., 2007). Cadherins mediate homotypic binding via the N-terminal domain (Pokutta and Weis, 2007), possibly prompting extrapolation of that idea to Als proteins. Donohue et al. (2011) produced soluble NT/T from Als1, immobilized the protein on a CM5 chip and used surface plasmon resonance to measure its interaction with itself. Results suggested homotypic binding, further supporting the conclusion that Als NT domains bind to each other. Lipke et al. (2012) postulated a model of Als homotypic binding and mechanical stimulus (provided by an atomic force microscopy probe) that resulted in the formation of cell-surface amyloid nanodomains (**Figure 3A**). A key feature of this model is binding of one Als NT domain to another.

**FIGURE 4:**
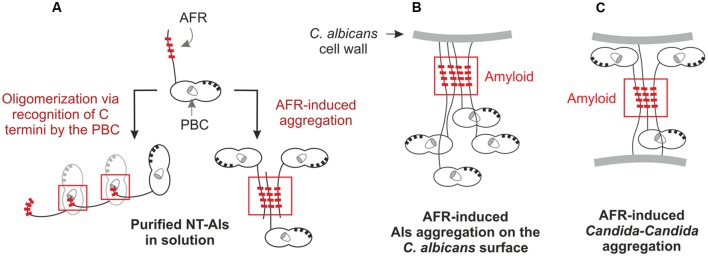
**Mechanisms of NT domain interactions between purified proteins **(A)** and between mature, full-length Als proteins on the *C. albicans* cell surface (B,C). (A)** Purified NT-Als proteins may interact by two mechanisms. The first involves PBC-mediated recognition of the free C-terminal peptide, leading to oligomerization of the NT-Als molecules (left). The second mechanism involves aggregation mediated by the AFR (right). Because the NT domain is a small portion of the full-length, mature Als protein, PBC-mediated oligomerization of the proteins cannot explain aggregation between Als molecules on the *C. albicans* cell surface. These interactions are more likely attributable to the AFR **(B)**. The AFR of mature, full-length Als proteins can also promote Als–Als-mediated aggregation between different *C. albicans* cells **(C)**.

While current structural data support the idea that Als NT domains bind to each other, it is necessary to distinguish between the type of interactions that occur when working with purified Als NT domains from those that are possible for mature, full-length Als proteins displayed on the *C. albicans* surface. NMR and X-ray crystallography data indicate two possible mechanisms for interaction of purified Als NT domains, as described previously for Als9-2 and Als3 (Salgado et al., 2011; Lin et al., 2014; **Figure 4A**). One mechanism involves recognition of the flexible C terminus of one NT domain by the PBC of another, leading to oligomerization of NT-Als proteins. The other mechanism involves aggregation of NT-Als proteins via exposed AFRs. Analysis of a shortened version of the Als NT (sNT-Als) showed that a flexible C terminus, including the AFR, is necessary for the observed interactions. Removal of the AFR from the NT structure, leaving only the N1 and N2 domains, eliminates the self-complementary binding, resulting in soluble monomeric protein, even at high concentration (Lin et al., 2014). For mature, full-length Als proteins on the *C. albicans* surface, the C terminus is anchored to the cell wall, leaving the intermolecular association of different AFRs as the only mechanism for interaction of Als NT (**Figure 4B**). This activity is consistent with the aggregative properties proposed for the AFR (Lipke et al., 2012; Lin et al., 2014).

The AFR has been the subject of considerable study. Lipke et al. (2012) communicated an overall vision for importance of the AFR in Als interactions. AFR-mediated interactions play a large role in formation of Als protein aggregates. Although this phenomenon could be called ‘adhesion,’ here we attempt to clarify molecular mechanisms by distinguishing aggregative interactions from those involving ligand binding. The AFR mediates aggregative interactions that cluster Als proteins together (amyloid nanodomains) on the fungal cell surface (**Figure 3A** and **4B**). Considerable data have been offered to support this conclusion (reviewed in Lipke et al., 2012). Mutagenesis of the AFR inhibits these interactions, suggesting that they are AFR-mediated.

**Figure 4C** extends the AFR model to demonstrate how interaction between Als AFRs on the surface of different cells may result in *C. albicans* aggregation. Presumably, these amyloid-driven interactions may also occur between Als AFRs and AFRs in other proteins, on the surface of *C. albicans* or other cell types. AFR-mediated interactions explain fungal aggregation over biological surfaces (i.e., host cells or bacteria). At present, though, it is unclear how these interactions would promote attachment to abiotic surfaces, especially those that are not coated in protein (discussed above). AFR-mediated interactions may explain some of the Als–Als interactions reported in the literature such as complementary function of Als proteins in biofilm formation (Nobile et al., 2008). Als protein interaction with the *C. albicans* cell-surface adhesin Hwp1 may also be AFR-mediated.

Studying an Als3 molecule with a mutagenized AFR in *C. albicans* demonstrated the complexities in dissecting phenotypes and attempting to ascribe mechanistic interpretations to data. The AFR of Als3 was mutagenized to replace Ile residues with Ser, thereby destroying amyloidogenic potential (I311S/I313S; Lin et al., 2014). The mutant strain (Als3-afr) was tested in standard *in vitro* adhesion assays. Interpretation of data at face value suggested that the AFR both increased and decreased *C. albicans* adhesion. These data illustrate the assay-dependency of the results and the need to dig deeper to reconcile the observations. Adhesion assays where *C. albicans* cells interacted with monolayers of human cells suggested that the AFR inhibited adhesion while assays that involved interaction with freshly collected human cells in a shaking flask suggested that the AFR promoted adhesion. One common observation was that the *C. albicans* cells in which Als3-afr was produced were less aggregated compared to the control strain. Results were interpreted to indicate that mutation of the AFR reduced aggregation of individual Als3 molecules on the *C. albicans* cell surface, freeing them to participate in adhesive interactions, rather than sterically hindering the PBC in a clump of Als3 proteins (**Figure 3**). Mutagenesis of the AFR also decreased aggregation between *C. albicans* cells, which lowered adhesion counts in the shaking flask assays. In these assays, any *C. albicans* that touches the mammalian cell is ‘adhesion-positive’ whether it is participating in a molecular interaction with the host cell surface, or just in contact because of its presence in a *C. albicans* aggregate. Overall results pointed to a role for the AFR in aggregation of Als3 molecules on the *C. albicans* surface, and its facilitation of formation of *C. albicans* multicellular aggregates. The literature on the function of the AFR is abundant, but to our knowledge no reports have been published so far describing the specific interaction of this region with a host cell or bacterial ligand.

Assays using purified protein also pointed to some other structural information regarding the AFR that has the potential to affect its function on the *C. albicans* surface (**Figure 3B**): the position of the AFR changes, depending on whether the PBC is in the ligand-bound or ligand-free form (Lin et al., 2014). In the ligand-free form, the AFR is dissociated from the surface of NT-Als3 and free to associate with other AFR sequences. When the NT-Als3 PBC binds a ligand, it undergoes a conformational change and the AFR becomes associated with the surface of the protein. This observation presents a novel opportunity to modulate Als3 activity because in the presence of higher-affinity ligands, it may be possible to shift the equilibrium of this reaction and decrease *C. albicans* aggregation. Decreased aggregation may have positive phenotypic effects such as making *C. albicans* cells more susceptible to the action of antifungal drugs. A higher-affinity ligand may serve as an anti-adhesion molecule, as well as the means to target drug delivery to the *C. albicans* surface.

Examples discussed in this section emphasize that Als proteins are multifunctional molecules with the potential to interact with other molecules or among themselves. Interactions among Als proteins can drive changes in the *C. albicans* surface or result in cellular aggregation that causes measurable differences in various phenotypic assays. Availability of NT-Als structural data has provided insight into the molecular mechanisms behind these interactions.

## Are Als Proteins Interchangeable?

This section addresses perhaps the most common question that arises when studying a protein family: are the various proteins interchangeable? In other words, can protein #1 replace the function of protein #2, suggesting they are functionally equivalent? Because Als proteins are a composite of many different functions (e.g., ligand binding, aggregation, attachment to abiotic surfaces), the answer may differ depending on which activity is considered. As detailed in the previous sections of this review, functions can be ascribed to different Als structural features. As we continue to dissect the Als molecule at the structural level, observations of functional equivalence can be used to derive new information such as identifying amino acids in the PBC that are responsible for ligand-binding specificity. Here, we examine published conclusions regarding functional equivalence and place them into a structural context.

Comparisons between the ligand-binding activity of Als1, Als3, and Als5 are the most useful literature observations relevant to the discussion of functional equivalence. The NT portion of the three proteins (amino acids 1–312 of the processed sequence) is 74% identical; Als1 and Als5 are 82% identical in this same region. Als1, Als3, and Als5 all bind to *Streptococcus gordonii* (Klotz et al., 2007; Silverman et al., 2010; Hoyer et al., 2014). Likewise, *S. cerevisiae* strains that overexpress *ALS1, ALS3*, or *ALS5* have similar ligand-binding profiles when tested against gelatin, fibronectin, laminin, epithelial, and endothelial cells (Sheppard et al., 2004). However, when the same set of strains is tested in a ferritin-binding assay, only Als3 is positive (Almeida et al., 2008). Using a diverse set of peptides, Klotz et al. (2004) demonstrated overlapping ligand-binding specificity for Als1 and Als5, but also demonstrated ligand-binding differences by identifying peptides that bound to one Als protein but not the other. Lin et al. (2014) identified fourteen amino acids in the NT-Als3 structure with side chains that interact with peptide ligands in the PBC. Eleven of these 14 amino acids are conserved in the PBC of NT-Als1; the three amino acids that are different may explain the ability of NT-Als3 to bind ferritin while NT-Als1 cannot. Comparisons between the 14 ligand-interacting amino acids in the PBC of NT-Als1 and NT-Als5 show four differences, which may account for the peptide-binding variation noted by Klotz et al. (2004). Collectively, these studies identify amino acids that could be mutagenized to demonstrate the structural features responsible for ligand-binding specificity.

Given the relatively large number of observations regarding Als ligand-binding function in the literature, it is surprising that there is little additional information that can be used in a discussion of Als protein functional equivalence. The sometimes-extreme allelic variability in the ALS family, coupled with a lack of detail regarding which allele or which portion of an Als protein was studied, conspire to complicate interpretation of published experiments. For example, two distinct forms of the NT domain of Als9 are known (named Als9-1 and Als9-2; 84% identical). Initial comparison of the proteins suggested that Als9-2 is more active in ligand binding than Als9-1 (Zhao et al., 2007a), so it is important to know which one was used in a specific experiment. There are also numerous sequence variants for the NT domain of Als5, leading to the potential for experimental results that are more or less similar to Als1 function (Zhao et al., 2007c). When testing mature proteins on the surface of a fungal cell, allelic variation in the numbers of copies of the TR sequence may also result in different functional conclusions (Oh et al., 2005). Finally, published manuscripts may not specify the portion of an Als protein that was studied. One common example is reference to the ‘N-terminal domain’ without an indication of whether this protein includes approximately 329 amino acids (now called NT) or 433 amino acids (NT/T; numbering based on the unprocessed protein sequence; **Figure 1A**). Understanding whether the assayed protein included the AFR and/or was competent for self-complementation is key to interpreting experimental results in a structural context.

There are few comments in the literature about functional equivalence outside of the peptide-binding activity for Als proteins. One could imagine, however, that an Als feature like the AFR might be complemented readily by a diverse set of Als proteins. We may also find that CT domains are widely interchangeable, especially if their function is limited to providing a structural stalk to project the remainder of the Als protein away from the *C. albicans* surface. Therefore, the answer to the question about Als interchangeability is likely to vary depending on which function is considered.

## Does Als Protein Abundance Affect Phenotypic Conclusions?

This question has the most straightforward answer of any asked in this review so far: yes. The relative abundance of Als proteins on the *C. albicans* cell surface can be evaluated using specific anti-Als monoclonal antibodies (reviewed in Cota and Hoyer, 2015). The quantity and localization of Als proteins varies naturally on the surface of wild-type *C. albicans* cells, providing disparate opportunities for the proteins to contribute to cellular phenotypes. In experimental constructs, Als protein levels may vary among strains and affect functional comparisons. Examples of the relationship between Als protein abundance and phenotype are discussed here.

Because of its generous quantities and widespread distribution on germ tubes (Coleman et al., 2009), Als3 is an ideal model for addressing the relationship between phenotype and protein abundance. A recent report described evaluating adhesive function of two *C. albicans* constructs that expressed the same, single *ALS3* allele (Lin et al., 2014). The first strain (1893; *Δals3/ALS3*) was constructed by deleting one wild-type *ALS3* allele. The other strain (3464) was constructed by integrating the *ALS3* allele into a *Δals3/Δals3* background. Immunolabeling with a monoclonal antibody specific for NT-Als3 showed less intense fluorescence for strain 3464 compared to 1893. Strain 3464 showed lower adhesion to mammalian cells compared to 1893, even though the strains displayed the same wild-type Als3 protein. Data were consistent with the conclusion that less cell-surface Als3 resulted in lower adhesive capacity in phenotypic assays. A similar conclusion was observed in a study of Als protein contributions to biofilm formation (Nobile et al., 2008). In that work, strains with reduced gene dosage showed a lower capacity to form biofilms.

Presumably, as the abundance of Als protein decreases, a point will be reached where activity cannot be detected, the assay will be interpreted as ‘negative,’ and the Als protein will be concluded to lack the assayed function. Conversely, experimental approaches that feature protein overproduction may create artifacts of high abundance. Because Als proteins can interact with each other and with other *C. albicans* surface proteins (detailed above), packing them too densely on the cell surface could lead to phenotypes that wild-type *C. albicans* would not produce. *C. albicans* has determined which levels of proteins are ‘just right’ and while experimentally manipulating the system, researchers struggle to reproduce this effect.

The concept of protein abundance can also contribute to the discussion of Als protein functional equivalence (discussed above in Section “ARE Als PROTEINS INTERCHANGEABLE?”) by explaining seemingly different phenotypic conclusions for very similar proteins. For example, did the adhesion assay produce a negative result because the Als protein cannot recognize the ligand or because there was not enough Als protein present for a measurable phenotype? Is one protein ‘better’ at mediating a particular function because its abundance and display more closely resemble wild-type levels or is the protein ‘better’ due to structural features that are not found in other Als proteins? Experimental controls that assess relative protein abundance are critical for accurate data interpretation.

Naturally low protein abundance for *C. albicans* Als7 has perhaps complicated efforts to determine whether the protein has adhesive function. At present, Als7 is the only Als protein in *C. albicans* for which adhesive function has not been documented. Attempts to assess adhesive function by overexpression failed to detect ligand binding, although cell-surface Als7 quantities could only be measured indirectly and appeared quite low (Sheppard et al., 2004). Deletion of *ALS7* in *C. albicans* led to increased adhesion of the mutant strain, an effect that still requires a molecular explanation (Zhao et al., 2007b). Study of Als7 is further complicated by a staggering number of allelic *ALS7* variants (Zhang et al., 2003), raising questions of whether assay results from a single allele would apply to them all. Recent structural solutions of the NT domain from three different Als proteins illustrated overall structure similarities that can be extrapolated to the remainder of the *C. albicans* Als family (Salgado et al., 2011; Lin et al., 2014). Among the Als proteins, Als7 has the largest amino acid variation in the PBC raising questions regarding its adhesive function. Ligand-binding analysis for Als7 might best be addressed using purified NT-Als7 and model peptides.

In contrast to Als7 that still lacks verification of adhesive function, published cell-biological experiments consistently demonstrate the importance of Als3 in many phenotypes including adhesion, biofilm formation and cellular invasion (Zhao et al., 2006; Phan et al., 2007; Nobile et al., 2008). It is unclear whether Als3 has unique structural features that allow it to perform these varied functions, or whether the relatively high Als3 abundance and widespread localization on the *C. albicans* cell surface simply provide greater functional opportunities for the protein.

Finally, it is worth noting that Als protein localization and abundance are different *in vitro* and *in vivo* (Coleman et al., 2009, 2012). Als1 localization on *C. albicans* cells recovered from *in vivo* animal models is more widespread than Als1 localization on cultured cells (Coleman et al., 2010). These immunolabeling observations likely explain other studies that demonstrated an *in vitro* biofilm formation defect for *C. albicans* strains lacking Als3, but wild-type biofilm for the same strain when tested *in vivo* (Nobile et al., 2006). More widespread distribution of Als1 *in vivo* promoted biofilm formation, even in an *als3/als3* mutant strain. Analysis of Als protein abundance and localization on *C. albicans* cells recovered from animal models and clinical cases is a rich area for additional studies.

## What Structural Feature(s) of Als Proteins Mediate(s) Invasion of Host Cells?

Invasion refers to the process of a microbe entering a host cell. The invasin is a protein that promotes this process. Als proteins were first proposed to have invasin function when Sheppard et al. (2004) noted that *S. cerevisiae* cells that produced Als1, Als3, or Als5 on the surface were taken up in low numbers by cultured human umbilical vascular endothelial cells (HUVEC). Subsequent work suggested that binding of Als proteins (particularly Als3) to cadherins promoted endocytosis by cultured HUVECs and oral epithelial cell lines (Phan et al., 2007). A coating of the Als1 NT/T domains was able to promote endocytosis of latex beads by FaDu (pharyngeal carcinoma) epithelial cells, but not by HUVECs, suggesting Als1 also has invasin function, but perhaps is less effective than Als3. Despite their high degree of sequence identity in the NT/T domains, Als3 appears to have superior invasin function compared to Als1 or Als5. Analysis of invasin function for other Als proteins has not been reported. It is possible that given sufficient abundance and cell-surface distribution, other Als proteins could demonstrate invasin function.

Wachtler et al. (2012) also studied *C. albicans* invasion of epithelial cells and evaluated the contributions of various proteins. Their work featured TR-146 cells (human squamous carcinoma of the buccal mucosa) and sought to separate the effects of induced endocytosis from those of *C. albicans* active penetration into the mammalian cells. Induced endocytosis involves rearrangement of the host cell actin cytoskeleton and *C. albicans* internalization (Phan et al., 2007). Active penetration is a more forceful process, which involves pushing the hyphal tip through the host cell membrane, often passing through multiple contiguous host cells (Wachtler et al., 2012). Analysis of *als3/als3* mutant strains, use of cytochalasin D to inhibit microfilaments and the induced endocytosis process, and elimination of active penetration by killing *C. albicans* germ tubes with thimerosal, were used as complementary approaches to demonstrate that active penetration is the main mechanism that *C. albicans* uses to invade TR-146 cells. Wachtler et al. (2012) demonstrated a role for Als3 in both induced endocytosis and active penetration.

Adhesion is one possible characteristic that Als3 needs for either induced endocytosis or active penetration. Both processes require *C. albicans* to be in close proximity (even intimate contact) with the host cell. Adhesion to host cell proteins is one way to mediate that contact. In the context of active penetration, Wachtler et al. (2012) postulated that Als3 adhesion provides a foothold for the *C. albicans* germ tube. This strong anchorage of *C. albicans* to the host cell permits the force needed for the germ tube tip to penetrate host cell membranes. The Als3 PBC would likely provide this interaction since mutation of the PBC (leaving a fully formed wild-type Als3 surface exposed on *C. albicans*) eliminates Als3 adhesive function (Lin et al., 2014). While it is easy to envision a role for adhesion in the invasion process, it is unclear if Als3 features other than the PBC are also involved. For example, Als3 may have surface features that promote host cell invasive interactions more efficiently than the surface features of Als1 or Als5. Comparison of the structures of these proteins would identify candidates for mutational analysis.

**FIGURE 5:**
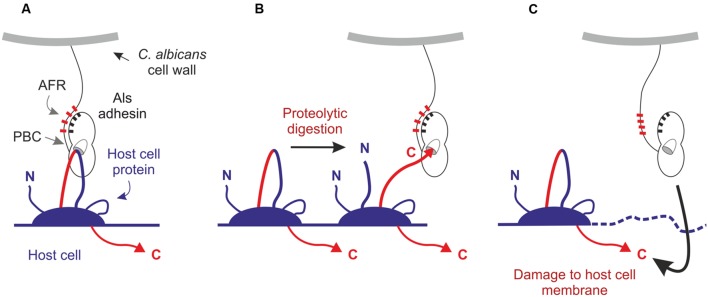
**Potential mechanisms to explain the PBC-mediated adhesive/invasive interactions of Als3 with host cells. (A)** The Als3 PBC may interact with extracellular features of intact cadherins or other mammalian cell-surface proteins. **(B)** *C. albicans* may release proteases to facilitate partial digestion of cell-surface proteins, producing free C termini that are anchored to the host-cell membrane and competent for interaction with the Als3 PBC. **(C)** *C. albicans* may damage the host-cell membrane and promote translocation of Als3 into the host-cell cytoplasm where it may contact the C termini of membrane-anchored proteins.

Invoking adhesion as an important contributor to invasion leads to the question of what proteins serve as Als3 binding partners. While numerous cell-surface proteins likely could interact with the Als3 PBC and provide a firm foothold for active penetration, cadherins were proposed to serve as the binding partner that promotes induced endocytosis (Phan et al., 2007). Work by Wachtler et al. (2012) supported this conclusion, but also identified an invasion mechanism that is independent of cadherin binding. Previous work demonstrated that Als3 ligand-binding function resides within the PBC and that the PBC prefers to bind free C-terminal peptides. Therefore, the Als3 PBC needs a way to contact the cadherin C terminus, which is located in the cytoplasm of the mammalian cell. These relationships set up an apparent contradiction and the need for alternative mechanisms to explain the ligand-binding interaction (**Figure 5**).

One possible explanation is that the Als3 PBC can bind other, yet undescribed, features on the extracellular portion of cadherins, or even bind other cell-surface proteins that promote invasion. A second possibility involves partial digestion of cadherins (Frank and Hostetter, 2007) by secreted proteases from *C. albicans* to generate extracellular C termini that remain anchored to the host cell membrane and become ligands for Als3. A third possibility is the translocation of Als3 through the host cell membrane as the initial event that leads to recognition of C termini. Damage of the cell membrane by *C. albicans* could promote exposure of cytoplasmic ligands for Als3.

Proposed explanations for published observations focus on Als3 PBC activity, but other features (such as the AFR) may also be involved in contacting host cell proteins and creating connections between fungal and mammalian cells. It is also helpful to note that published observations involve different cell lines and different cell types, and it is possible that mechanistic details for *C. albicans* invasion may vary among them. Adhesion and invasion assays using Als3 mutants produced in *C. albicans* and host cell lines with engineered cadherin molecules will clarify these relationships and provide the tools needed for detailed structural analyses of Als/cadherin complexes.

## Epilogue

Considerable progress has been made toward understanding the composition of the *C. albicans* ALS family and the function of its encoded proteins. Mechanistic explanations for Als protein function were elusive until recently and have been advanced by the availability of detailed NT-Als structural data. These data promote clarity in descriptions of Als function because function can be ascribed to specific structural features and precise words can be selected to describe the various interactions that Als proteins mediate. Because Als proteins are large molecules, many features remain to be examined at the structural level and placed into a functional context. Future investigations will also focus on understanding the boundaries of the ALS family and which genes from other species merit inclusion. Work in *C. albicans* provides the foundation for these more extensive explorations. As it has from the beginning, the ALS family provides a fertile area of inquiry with many fascinating questions to answer.

## Author Contributions

LH and EC developed and wrote the manuscript. Each contributed to design and construction of the figures and table.

## Conflict of Interest Statement

The authors declare that the research was conducted in the absence of any commercial or financial relationships that could be construed as a potential conflict of interest.

## Abbreviations

AFR: amyloid-forming region
Als: agglutinin-like sequence
CSH: cell surface hydrophobicity
CT: Als C-terminal domain
NT or NT-Als: Als N-terminal domain
PBC: peptide-binding cavity
T: Als Thr-rich domain
TR: Als tandem repeat domain
